# STAMP2 is required for human adipose-derived stem cell differentiation and adipocyte-facilitated prostate cancer growth *in vivo*

**DOI:** 10.18632/oncotarget.11131

**Published:** 2016-08-09

**Authors:** Torstein Lindstad, Su Qu, Jørgen Sikkeland, Yang Jin, Alexandr Kristian, Gunhild M. Mælandsmo, Philippe Collas, Fahri Saatcioglu

**Affiliations:** ^1^ Department of Biosciences, University of Oslo, Oslo, Norway; ^2^ Department of Cancer Genetics and Informatics, Oslo University Hospital, Oslo, Norway; ^3^ Department of Tumor Biology, Institute for Cancer Research, Oslo University Hospital, Oslo, Norway; ^4^ Institute of Basic Medical Sciences, Norwegian Center for Stem Cell Research, University of Oslo, Oslo, Norway

**Keywords:** STAMP2, ASC, lipogenesis, adipogenesis, prostate cancer

## Abstract

Six Transmembrane Protein of Prostate 2 (STAMP2) has been implicated in both prostate cancer (PCa) and metabolic disease. STAMP2 has unique anti-inflammatory and pro-metabolic properties in mouse adipose tissue, but there is limited information on its role in human metabolic tissues. Using human adipose-derived stem cells (ASCs), we report that STAMP2 expression is dramatically upregulated during adipogenesis. shRNA-mediated STAMP2 knockdown in ASCs significantly suppresses adipogenesis and interferes with optimal expression of adipogenic genes and adipocyte metabolic function. Furthermore, ASC-derived adipocyte-mediated stimulation of prostate tumor growth in nude mice is significantly reduced upon STAMP2 knockdown in ASC adipocytes. These results suggest that STAMP2 is crucial for normal ASC conversion into adipocytes and their metabolic function, as well as their ability to facilitate PCa growth *in vivo*.

## INTRODUCTION

Obesity has become a global epidemic estimated to affect more than one billion people worldwide by 2030 [1]. Continued energy overload increases the number (adipogenesis) and size (lipogenesis) of triglyceride-storing adipocytes. Eventually, however, the adipose tissue can no longer store more fat. As a result, systemic triglyceride levels increase and dramatically accelerate development of obesity-associated pathologies, such as type 2 diabetes, atherosclerosis and some types of cancer [2, 3].

Adipogenesis is the process in which progenitor, fibroblast-like cell types differentiate into adipocytes, and is probably one of the best-understood developmental processes *in vitro* [4]. In addition to adipocytes, adipose tissue contains other cell types, such as immune cells, fibroblasts, endothelial cells, mesenchymal stem cells and ASCs, all with critical functions for adipose tissue development, homeostasis and function [5]. ASCs share high similarity to mesenchymal stem cells as they both can be differentiated into the same types of cell types, express common cell surface markers, and share similar transcriptomes [6, 7]. Nevertheless, a homogenous population of CD34+, CD105+, CD31-, and CD45- cells is unique and represents ASCs that can readily be isolated from human lipo-aspirate material and differentiated into all mesodermal cell types *in vitro*, including adipocytes and osteocytes [8, 9].

Recent work has implicated obesity in the development and progression of some cancer types including those affecting the oesophagus, colon, rectum, kidney, pancreas, breast, ovary, and endometrium [10–12]. There are mixed findings on the association between adipose tissue and PCa incidence, but there is accumulating evidence indicating that obesity is associated with an increased risk of advanced disease or death from PCa [13]. For example, a recent study found a more than 2-fold increase in PCa recurrence in obese men when analysis was limited to non-diabetics [14]. Another recent study reported that obesity is linked with poorer PCa prognosis primarily in men with tumors harboring the gene fusion TMPRSS2:ERG [15]. Several mechanisms have been suggested to link obesity and lethal PCa, such as increased aromatase activity, dysregulated adipokine secretion, and increased secretion of vascular endothelial growth factor, cytokines and prostaglandins [13, 16]. However, the exact molecular mechanisms as to how each factor and their interplay with other factors/signaling pathways affect the link between obesity and PCa is currently not understood.

The STAMP protein family consists of three highly similar metalloreductases, STAMP1-3. It is also known as Six Transmembrane Epithelial Antigen of the Prostate (STEAP) family, which includes STEAP1 that lacks the N-terminal domain present in STAMP1-3. STAMPs were originally discovered for their possible role in carcinogenesis and have also been implicated in cell differentiation, proliferation, and apoptosis (for a review, see [17]). STAMP2, also known as STEAP4 and tumor necrosis factor-α-induced adipose-related protein (TIARP), is up-regulated by several pro-inflammatory cytokines in both murine and human metabolic tissue [18–26]. In addition, STAMP2 expression is induced during adipocyte differentiation [18–21, 27–29]. Furthermore, *in vitro* suppression of Stamp2 expression in 3T3-L1 cells inhibits adipogenesis [20]. Interestingly, murine Stamp2 expression is dysregulated in metabolic tissue of obese and diabetic animal models [19, 25, 30–32] and plays an important role in the regulation of systemic metabolic homeostasis in mice [19, 31, 32]. In humans, STAMP2 seems to play a similar role, but the exact picture is currently less clear. STAMP2 expression is dysregulated in patients with obesity and/or metabolic syndrome; however, STAMP2 expression was found to be both upregulated [33, 34], and downregulated [26, 27, 32] in these conditions. Furthermore, a STAMP2 gene polymorphism has been associated with metabolic syndrome in Han-Chinese and Hispanic populations [35, 36]. In addition, *in vitro* studies have linked STAMP2 to insulin sensitivity and glucose uptake in human hepatocytes and adipocytes [32, 37–39].

In this study, we investigate STAMP family gene expression during adipogenic differentiation from ASCs. We establish ASCs with stable suppression of STAMP2 and assess the effect of this on adipogenic conversion and the expression of signature genes involved in adipogenesis, as well as adipocyte metabolic function. Furthermore, we use this system to investigate the potential role of STAMP2 in adipocyte-facilitated PCa growth *in vivo*.

## RESULTS

### STAMP2 expression is upregulated during adipose stem cell differentiation

Several studies have shown that STAMP2 expression is greatly induced upon adipocyte differentiation *in vitro* [18–21, 27–29]. We have recently also shown that murine *Stamp1* and *Stamp3* mRNA expression is regulated in 3T3-L1 cells upon their adipogenic conversion [20].

**Figure 1:**
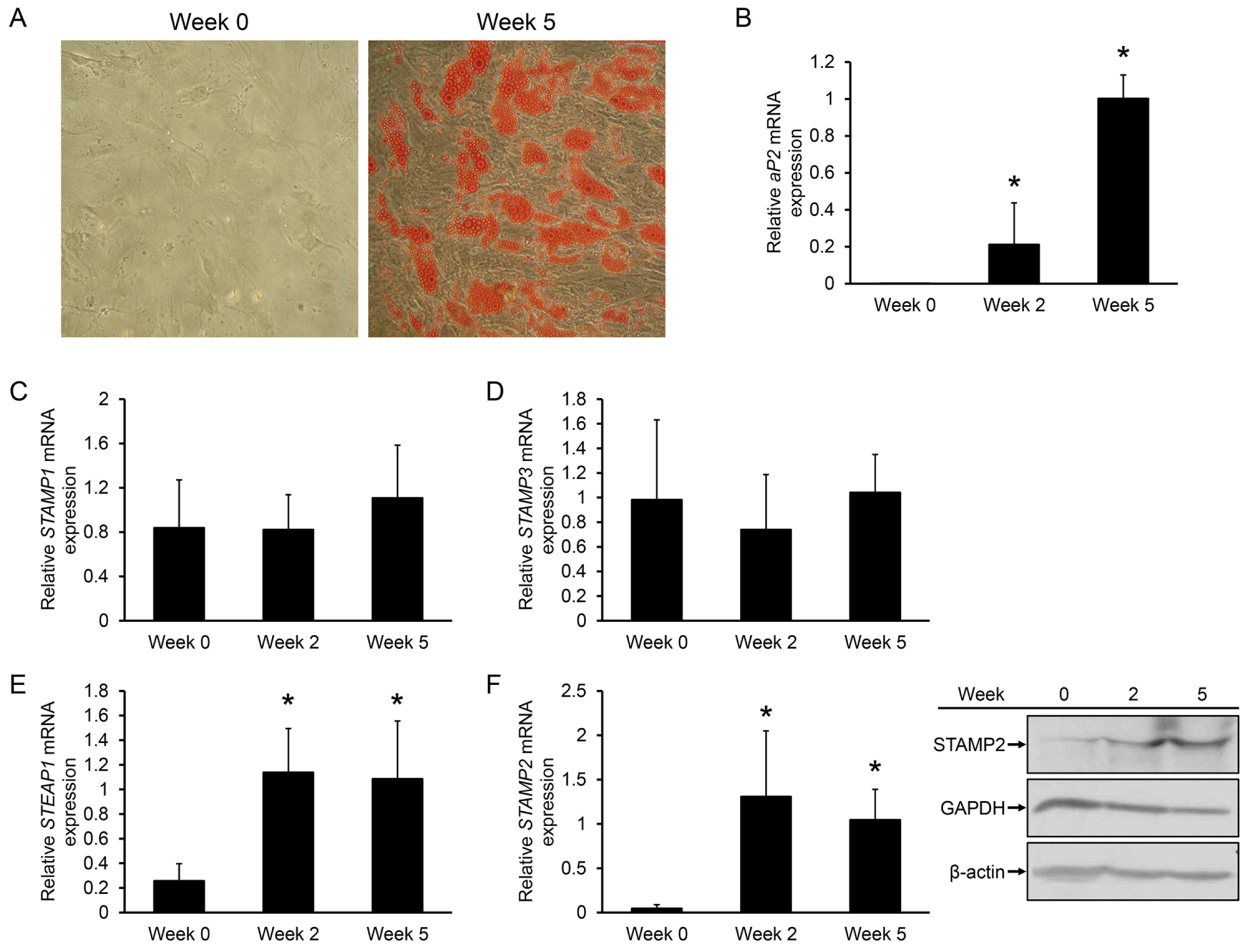
Regulation of STAMP family expression during ASC adipogenesis **A.** Oil Red O staining of undifferentiated ASCs and ASCs induced to differentiate into adipocytes for 5 weeks. **B-F.** qRT-PCR analysis of ASCs harvested at the indicated time points of differentiation. The figures show the mRNA expression of *aP2* (B), *STAMP1* (C), *STAMP3* (D), *STEAP1* (E), and *STAMP2* (F, left) normalized to the reference gene *GAPDH*. The results are from three independent experiments, n = 10. ^*^p<0.05 compared to week 0. (F, right) Western analysis showing STAMP2, GAPDH, and β-actin protein levels from ASCs harvested in parallel to those used for qRT-PCR analysis.

To investigate if STAMP expression is regulated during adipogenesis of human cells, human ASCs were isolated from lipo-aspirates, plated on plastic dishes and incubated with an adipogenic cocktail. After approximately 2 weeks, small lipid droplets started to accumulate (data not shown) and after 5 weeks approximately 50% of the cells had lipid droplets readily visualized by Oil Red O staining (Figure 1A). Gene expression analysis confirmed adipogenic conversion: the adipocyte marker fatty acid binding protein 4 (FABP4/aP2) was not expressed in undifferentiated ASCs, but was highly expressed at 2 weeks, and continued to increase at 5 weeks after induction of differentiation (Figure 1B).

We then assessed possible changes in *STAMP* and *STEAP1* expression during ASC differentiation. qRT-PCR analysis showed that *STAMP1* and *STAMP3* expression did not significantly change upon ASC adipogenic conversion (Figure 1C and 1D). In contrast, *STEAP1* and *STAMP2* expression which were low in undifferentiated ASCs, markedly increased (about 4.5-fold and 30-fold, respectively) after 2 weeks of differentiation and remained high after 5 weeks (Figure 1E and 1F). Similar results were obtained for STAMP2 at the protein level (Figure 1F, right). These results are consistent with that seen in differentiation of murine 3T3-L1 preadipocytes [18–20], and with previous studies using ASCs or human preadipocytes [27, 28].

### STAMP2 knockdown interferes with adipogenesis and lipogenesis of ASCs

**Figure 2:**
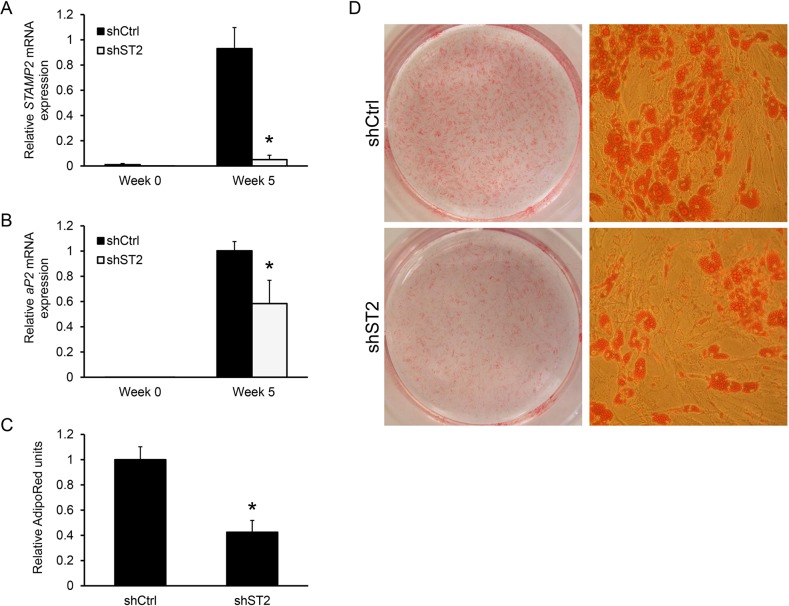
Knockdown of STAMP2 suppresses ASC adipogenesis **A-B.** ASCs expressing a non-silencing shRNA (shCtrl) or shRNA against STAMP2 (shST2) were harvested at the indicated time points of differentiation and subjected to qRT-PCR analysis. The figures show the mRNA expression of *STAMP2* (A) and *aP2* (B) normalized to the reference gene *GAPDH*. The results are from three independent experiments, n = 10. ^*^p<0.05 comparing shST2 to shCtrl. **C.** AdipoRed assay with differentiated ASC shCtrl and ASC shST2 (Week 5). **D.** Oil Red O staining of differentiated ASC shCtrl and ASC shST2 (Week 5). The images are representative for three independent experiments.

Given the dramatic increase in STAMP2 expression during ASC differentiation, as well as similar expression pattern and importance of murine Stamp2 for *in vitro* adipogenesis of 3T3-L1 cells [20], we determined whether STAMP2 may be involved in this process. Undifferentiated ASCs were transduced by a lentivirus expressing either a shRNA targeting *STAMP2* or non-silencing control shRNA. At 5 weeks of adipogenic induction, *STAMP2* expression was decreased by >90% in STAMP2 knockdown cells compared with control cells (Figure 2A). STAMP2 knockdown led to significantly lower expression of *aP2* (Figure 2B) and resulted in decreased lipid accumulation as shown by AdipoRed assay and Oil Red O staining (Figures 2C and 2D, respectively). These data show that suppression of *STAMP2* expression interferes with the *in vitro* differentiation of human ASCs.

**Figure 3:**
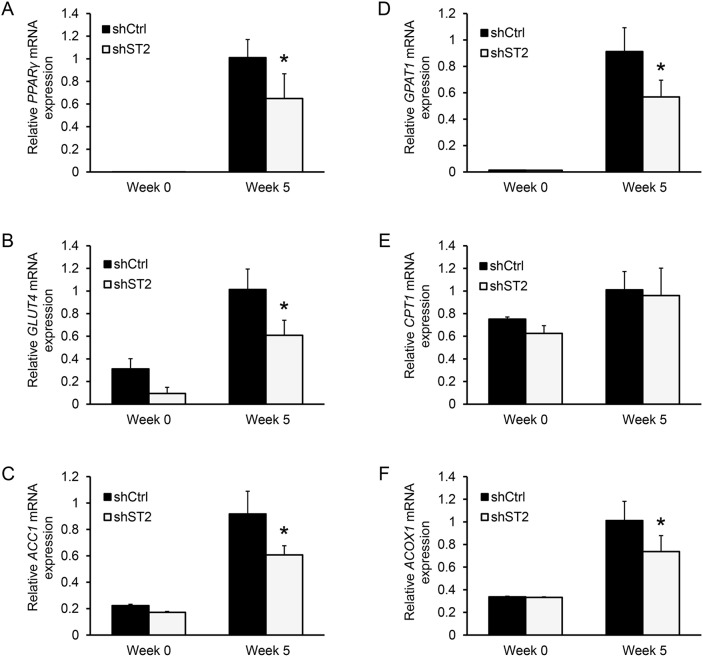
Knockdown of STAMP2 affects the expression of genes involved in energy uptake, lipid synthesis, and storage **A-F.** ASCs expressing non-silencing shRNA (shCtrl) or shRNA against STAMP2 (shST2) were harvested at the indicated time points of differentiation and subjected to qRT-PCR analysis. The figures show the mRNA expression of *PPARγ* (A), *GLUT4* (B), *ACC1* (C), GPAT1 (D), *CPT1* (E), and *ACOX1* (F) normalized to the reference gene *GAPDH*. The results are from three independent experiments, n = 10. ^*^p<0.05 comparing shST2 to shCtrl.

We have previously shown that Stamp2 suppression in 3T3-L1 cells inhibits adipogenesis in concert with downregulation of transcription factors essential for adipocyte development and function: CCAAT/enhancer binding protein alpha (C/EBPα) and peroxisome proliferator-activated receptor gamma (PPARγ) [20]. In agreement with our previous findings, *PPARγ* expression was downregulated in ASC adipocytes upon STAMP2 knockdown compared to control cells (Figure 3A). PPARγ is critical for adipocyte function, including regulating energy uptake and storage [40]. We thus assessed the expression of PPARγ target genes in STAMP2 knockdown cells. Among these PPARγ targets, *Glucose transporter 4* (*GLUT4*), the main channel through which glucose is imported into adipocytes [41], was downregulated (Figure 3B). Similar results were obtained for *Acetyl-CoA carboxylase 1* (*ACC1*) (Figure 3C) which catalyzes the irreversible carboxylation of acetyl-CoA to produce malonyl-CoA, the first step in fatty acid (FA) biosynthesis [42]. *Glycerol-3-phosphate acyltransferase 1* (*GPAT1*), which catalyzes the rate limiting step in the formation of triacylglycerols (TGs) from FAs was also downregulated (Figure 3D) [43]. In contrast, *Carnitine palmitoyl transferase-1* (*CPT1*) expression was not affected (Figure 3E). CPT1 is involved in the transport of long FAs across the mitochondrial membrane for β-oxidation [44] and compete with GPAT1 [42]. Moreover, the expression of *peroxisomal acyl-coenzyme A oxidase 1* (*ACOX1*), which catalyzes the first step in very-long FA catabolism, was downregulated (Figure 3F) [45]. In summary, these data indicate an overall impaired function of energy uptake, lipid synthesis, and lipid storage of ASC-derived adipocytes upon suppression of *STAMP2* expression.

### STAMP2 is required for adipocyte induced prostate tumor growth in a preclinical model

**Figure 4:**
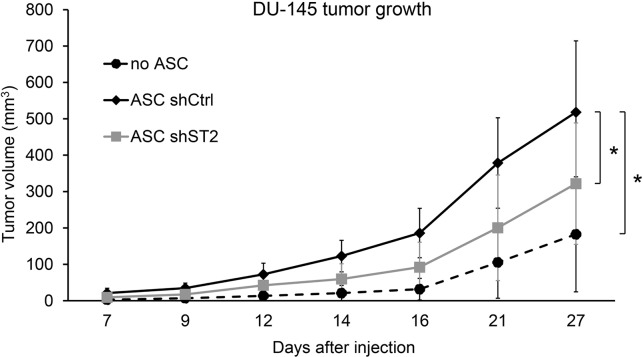
STAMP2 knockdown attenuates ASC-derived adipocyte stimulation of DU-145 tumor growth Tumor volumes from xenografts of DU-145 alone and DU-145 co-injected with ASCs expressing non-silencing shRNA (shCtrl) or shRNA against STAMP2 (shST2) were measured at the indicated time points. The results are from one xenograft study with 4 animals bearing 2 tumors each (n=8 per group). ^*^p<0.01 comparing the indicated tumor groups for all time points.

Previous studies have shown that adipose-derived stem cells have stimulatory effects on prostate tumor growth in immunodeficient mouse models [46, 47]. To determine if STAMP2 expression in ASCs affect their ability to stimulate PCa development, DU-145 PCa cells were injected subcutaneously into nude mice either alone or in the presence of differentiated ASCs expressing either non-silencing shRNA controls or shRNA targeting STAMP2. Tumor formation was then monitored over time. In the presence of ASC-derived adipocytes DU-145 cells formed tumors that were significantly larger than tumors that formed by DU-145 cells alone (Figure 4). In contrast, ASCs in which STAMP2 expression was knocked down significantly lost their ability to stimulate DU145 tumor growth compared with control cells (Figure 4). These data show that STAMP2 is required for stimulation of PCa growth by ASC-derived adipocytes.

## DISCUSSION

In this study we have investigated possible changes in STAMP expression during ASC differentiation into adipocytes. There was no significant change in *STAMP1* or *STAMP3* expression, which differ from previous findings in murine cells [20, 48, 49]. In contrast, *STEAP1 and STAMP2* levels increased throughout the adipogenesis process. In murine bone marrow derived mesenchymal stem cells, *STEAP1* was earlier reported to be downregulated upon differentiation into the adipogenic lineage [48]. The discrepancy with our findings may represent differences in the role these proteins play in adipogenesis in a murine or human context. Alternatively the divergent results may be due to the differences in the stemness of the cells used in the experiments. The marked up-regulation of *STAMP2* expression is in agreement with previous reports from human cells using ASCs and isolated pre-adipocytes [27–29], as well as with several reports from murine models [18–21]. Taken together, these data suggest that STAMP2 expression is a hallmark of mammalian adipocyte differentiation.

Our results also show that STAMP2 is required for efficient adipogenesis *in vitro* as suppression of its expression led to reduced adipogenic conversion of ASCs. This is in agreement with our recent findings that Stamp2 also regulates this process in murine 3T3-L1 cells [20]. The genetic and morphological development of adipocytes are under the control of PPARγ [50]. Similar to what we have shown in murine cells, *in vitro* STAMP2 knockdown negatively affected PPARγ expression [20]. Some of the many transcriptional targets of PPARγ are genes involved in energy uptake and storage [40]. Consistently, *GLUT4*, *ACC1*, *GPAT1*, and *ACOX1* are all downregulated in differentiated ASCs. These adipocytes will have less potential to import glucose through GLUT4. Inhibition of GLUT4 expression or function has been reported in several adipocyte systems where dysregulation of STAMP2 has been induced [19, 24, 37, 39]. Inhibited glucose uptake ultimately means less available acyl-coenzyme A as substrate for an already downregulated ACC1 enzyme for conversion into malonyl-CoA for subsequent FA synthesis. In addition to decreased *de novo* FA synthesis, peroxisomal breakdown of very-long to long FAs is controlled by ACOX1. These two sources of FA are substrates for the first step to store energy as TG governed by GPATs. Interestingly expression of CPT1, transporter of long FAs into the mitochondria destined for β-oxidation is not affected by STAMP2 knockdown. Collectively, this suggests that STAMP2 knockdown adipocytes have decreased ability to obtain and store energy during feeding, and subsequent less energy to distribute in periods of fasting.

Obesity potentially influences cancer development through several axes with both local and systemic action: insulin/insulin growth factor signaling, dysregulation of sex hormones and adipokines, and inflammation (for reviews, see [13, 51]). We used an *in vivo* experimental system that mimics increased (periprostatic) adipocyte levels in the local tumor environment, and observed that tumors formed upon co-injection of DU-145 cells and ASC adipocytes were significantly larger than those generated by DU-145 cells alone. Our findings are in agreement with previously reported experimental systems testing various features in which adipocytes affect PCa growth: co-culture of PC-3 cells with rat epididymal adipocytes increased intracellular lipid content in cancer cells and stimulated their proliferation [52]. *In vitro* culture of PCa cells in the presence of adipocyte conditioned media (CM) was enough to promote increased proliferation and motility [53–56]. The critical factors present in the adipocyte CM were shown to be monocyte chemotactic protein-1 (MCP-1) [54], CC chemokine ligand 7 (CCL7) [56], and matrix metalloproteinases (MMPs) 2 and 9 [53, 54]. Of note, conditioned media from PC-3 cells stimulated the expression of a range of adipokines and cytokines such as leptin, IL-6, and TNFα in adipose tissue explants [57]. In addition, MMP2 and MMP9 activity was increased. This emphasizes the bilateral communication taking place in the tumor micro-environment between the tumor cells and the adipocytes.

aP2 is a novel adipokine that was earlier found to facilitate lipid transfer from adipocytes to ovarian cancer cells promoting growth in *in vitro* co-culture [58, 59]. In both ASCs and murine 3T3-L1 adipocytes suppression of STAMP2 leads to a decrease in *aP2* expression [20]. consistent to our findings in ASCs. aP2 directly promoted *in vitro* PCa cell growth and was essential for oleic acid stimulation of PCa cell invasion [60]. Furthermore, *in vivo* pharmacological inhibition of aP2 decreased DU-145 tumor growth in mice [60]. Apart from playing a role in facilitating oleic acid signaling, one mode of action of aP2 was linked to activation of phosphoinositide 3-kinase (PI3K) and protein kinase B (PKB/Akt), signaling pathways that have previously been implicated in PCa [61].

We have previously shown that STAMP2 promotes PCa growth [62, 63]. Here we provide evidence for an additional role of STAMP2 in PCa growth through its role in at least one cell type in the tumor micro-environment, ASCs. Since STAMP2 is important for the expression of genes whose products are central in energy uptake, transport, and storage, this may be necessary for providing the optimal growth conditions for tumor cells. Further work is required to uncover the molecular mechanisms of how STAMP2 expression in the microenvironment can drive PCa growth.

## MATERIALS AND METHODS

### Cell culture

Adipose tissue was obtained by liposuction from abdominal, hip, and thigh regions of healthy female donors after formal consent. Stromal vascular portion was used for isolation of ASCs essentially as previously described [8]. Briefly: lipoaspirate (300–400 ml) was washed and digested with 0.2% collagenase (Sigma-Aldrich) for 2 h at 37°C with shaking. Floating adipocytes were separated from the stromal vascular fraction by centrifugation. After lysis of erythrocytes and sedimentation, the cellular pellet was resuspended and strained successively through 100- and 40-μm sieves. Magnetic beads were then used to remove CD45^+^ and CD31^+^ cells, and as previously reported, the remaining cell population expressed a CD45^−^CD31^−^CD34^+^CD105^+^ phenotype [8]. Immediately after separation, cells were washed and resuspended in DMEM/F12 (LONZA) supplemented with 20% fetal bovine serum (FBS, Saveen Werner), Pen-Strep (LONZA), and 2.5 μg/ml amphotericin B (Sigma-Aldrich). After 7 days in culture, attached cells were passaged by trypsinization and cultured further in DMEM/F12 supplemented with 10% FBS and antibiotics. ASCs were cultured and passaged at 70–80% confluency.

### Adipogenic differentiation

The polyclonal ASC lines obtained as described above were cultured to confluence before differentiation. For adipogenic differentiation, confluent ASCs were cultured in DMEM/F-12 with 10% FBS and stimulated for up to 5 weeks with 0.5 mM 1-methyl-3 isobutylxanthine (Sigma-Aldrich), 1 μM dexamethasone (Sigma-Aldrich), 10 μg/ml insulin (Sigma-Aldrich), and 200 μM indomethacin (Sigma-Aldrich). Medium was changed every 3-4 days.

### Oil Red O staining

The cells were washed briefly with PBS and then fixed with 0.5% gluteraldehyde in PBS followed by washes with PBS and 60% isopropanol (Arcus) in PBS. The cells were then stained in Oil Red O solution (3 parts Oil Red O [0.5 g (Sigma-Aldrich in 200 ml isopropanol] and 2 parts MQ water) for 15 min and washed with 60% isopropanol followed by a final wash in PBS.

### AdipoRed assay

Performed using LONZA product protocol: Washed cells with PBS, and added AdipoRed. Incubated for 15 min and measured the fluorescence (excitation 485 nm, emission at 572 nm) using a plate reader (Victor2, PerkinElmer).

### Stable lentiviral shRNA cell lines

The pGIPZ lentiviral shRNAmir construct (Open Biosystems) containing the shSTAMP2 target sequence: 5′-CGC CAA GAA GTC TGA CAT CAT ATA GTG AAG CCA CAG ATG TAT ATG ATG TCA GAC TTC TTG GCT-3′ or a non-silencing-GIPZ lentiviral shRNAmir control sequence was transfected into the HEK293 packaging cell line together with packaging plasmids using the transfection agent FuGENE 6 (Roche) following the suppliers’ suggested protocols. Expression of lentiviral constructs was confirmed by detection of GFP by confocal microscopy (Olympus). 2 ml of lentivirus containing medium was incubated with ASCs for 48 hours. Cells were then grown under selection pressure with 2 mg/ml puromycin (Invitrogen) for 10 days. Successful transduction was confirmed by detection of GFP positive cells using confocal microscopy (Olympus). RT-qPCR analysis of markers for off-target RNAi effects in these cell lines showed that their expression levels were not altered (Supplementary Figure S1B-S1E).

### Quantitative Reverse-transcription PCR (qRT-PCR)

Total RNA was extracted from cells using the Trizol reagent (Invitrogen). mRNA transcripts were converted to cDNA by the Superscript II (Invitrogen) reverse transcriptase using oligo(dT) primers (Sigma-Aldrich). cDNA was quantified by the Lightycler480 system using the SYBR Green dye (Roche). For each primer pair the crossing point (CP) values of a given PCR for a sample were set relative to the CP value of the control group, while also correcting for primer specific reaction efficiency with an internal standard curve. The values were then normalized to the expression of the reference gene *GAPDH*. All PCR products were analyzed by melting curve analysis. qRT-PCR primer sequences (all from Sigma-Aldrich) used in this study are as follows: *aP2*, forward 5′-TAC TGG GCC AGG AAT TTG AC-3′, reverse 5′-TGG TTG ATT TTC CAT CCC AT-3′; *STEAP1,* forward 5′-TTT GGT GCA AAT GCA AAA gc-3′, reverse AGG GTC AAG CTA AGG CGA AG-3′; *STAMP1*, forward 5′-GCT CTT GTT TTG CCC TCA AT-3′, reverse 5′-GGG GAG ACA TGA GGA ATT GTT-3′; *STAMP2*, forward 5′-ATG ACA GCA AAG CCA AGC AA-3′, reverse 5′-GCA AAG CAT CCA GTG GTC AA-3′; *STAMP3*, forward 5′-GAG CAC ACT GCA CAC GCT CA-3′, reverse 5′CTC CCT CTC CCA GCC CTC TCC-3′; β-actin, forward 5′-GGC TAC AGC TTC ACC ACC AC-3′, reverse 5′-GTC AGG CAG CTC GTA GCT CT-3′; *GAPDH*, forward 5′-GTC AGT GGT GGA CCT GAC CT-3′, reverse 5′-GTC AGT GGT GGA CCT GAC CT-3′; *GLUT4*, forward 5′-CAG ATC GGC TCT GAC GAT G-3′, reverse 5′-ACT GAA GGG AGC CAA GCA C-3′; *PPARg*, forward 5′-TCA AGA CAA CCT GCT ACA AGC CCT, reverse 5′-AAG AAG GGA AAt GTT GGC AGT GGC-3′; *ACC1*, forward 5′-GTT GCA CAA AAG GAT TTC AG-3′, reverse 5′-CGC ATT ACC ATG CTC CGC AC-3′; *GPAT1*, forward 5′-GAT GGC TTG CAA GAC GCC TTT CTT-3′, reverse 5′-GCC ACT TCT GCA ATT GCC TCT TGT-3′; *CPT1*, forward 5′-TGG AGT CCC CTT TCC TTA AG-3′, reverse 5′-CCG TCA TCA GCA ACC G-3′; *ACOX1*, forward 5′-GCT AAG AAC TCC CCA CTG AA-3′, reverse 5′- GAC ACT TCA GAG CTT GGA C -3′; *IFIT1*, forward 5′-TTG CCT GGA TGT ATT ACC AC-3′, reverse 5′-GCT TCT TGC AAA TGT TCT CC-3′; *IFIT3*, forward 5′-GAA CAT GCT GAC CAA GCA GA-3′, reverse 5′-CAG TTG TGT CCA CCC TTC CT-3′; *MX1*, forward 5′-AGG ACC ATC GGA ATC TTG AC-3′, reverse 5′-TCA GGT CCA ACA CGA GGT TC-3′; *XPO5*, forward 5′-TGT TAA CCC GAG AAG TCA TGG-3′, reverse 5′-GGT CTG TAA GCT CTG CCA TAG-3′;

### Western analysis

ASCs were washed with PBS and whole cell extracts were prepared by resuspending cell pellets in 100 μl lysis buffer (20 mM HEPES (pH 7.7) [Sigma-Aldrich], 300 mM NaCl [Sigma-Aldrich], 1.5 mM MgCl2 [Sigma-Aldrich], 0.2 mM EDTA [Sigma-Aldrich], 0.1% Triton X-100 [Sigma-Aldrich], 0.5 mM dithiothreitol [Sigma-Aldrich] and a cocktail of protease inhibitors [Roche]) and incubated at 4°C for 1 hour. The lysate was then centrifuged at 13,000g for 15 min at 4°C to remove cell debris. 50 mg of denaturated protein extract was resolved on a 10% polyacrylamide (Sigma-Aldrich) SDS-gel, transfered to a PVDF membrane (BioRad), and incubated with STAMP2 antiserum (Abcam, 1:1000) in TBS+0.1% Tween, or with GAPDH antiserum (Santa Cruz, 1:1000) or β-actin antiserum (Santa Cruz, 1:1000) in 5% BSA TBS+0.1% Tween (Sigma-Aldrich). Secondary antibodies used: horseradish peroxidise-conjugated (HRP) α-mouse IgG antibody (1:5000, Sigma), HRP α-rabbit IgG antibody (1:10000, Sigma). Immunoreactive bands were detected using ECL Western blotting reagents (GE Healthcare) and images were obtained using a film developer (Optimax, Protech).

### Xenograft experiment

The ASCs expressing shRNA against STAMP2 or a non-silencing control were differentiated into adipocytes, counted, collected, and mixed with DU-145 cells. The cell mixes were then subcutaneously implanted into both flanks of male nude mice (four mice per group). 5 million DU-145 cells were injected per site in DU-145 only group, 5 million DU-145 cells and 0.5 million of the ASCs were injected per site for the other groups. Tumors were measured with a caliper at the indicated time points and the volumes were calculated using the formula: volume = (width × width × length)/2.

### Statistics

Statistical analyses were performed using the Student's t-test (Figure 1-3), and two-way ANOVA with Tukey's post-hoc test (Figure 4). Data are presented as means and error bars represent standard deviation.

## SUPPLEMENTARY MATERIALS FIGURE


