# Supplementary material for: STAMP2 is required for human adipose-derived stem cell differentiation and adipocyte-facilitated prostate cancer growth in vivo
Source: Oncotarget. 2016 Aug 9;8(54):91817–27. doi: 10.18632/oncotarget.11131 (PMC5696144; doi:10.18632/oncotarget.11131)
Supplement: Supplementary file 1 [file oncotarget-08-91817-s001.pdf]

STAMP2 is required for human adipose-derived stem cell differentiation and adipocyte-facilitated prostate cancer growth *in vivo*

SUPPLEMENTARY FIGURE

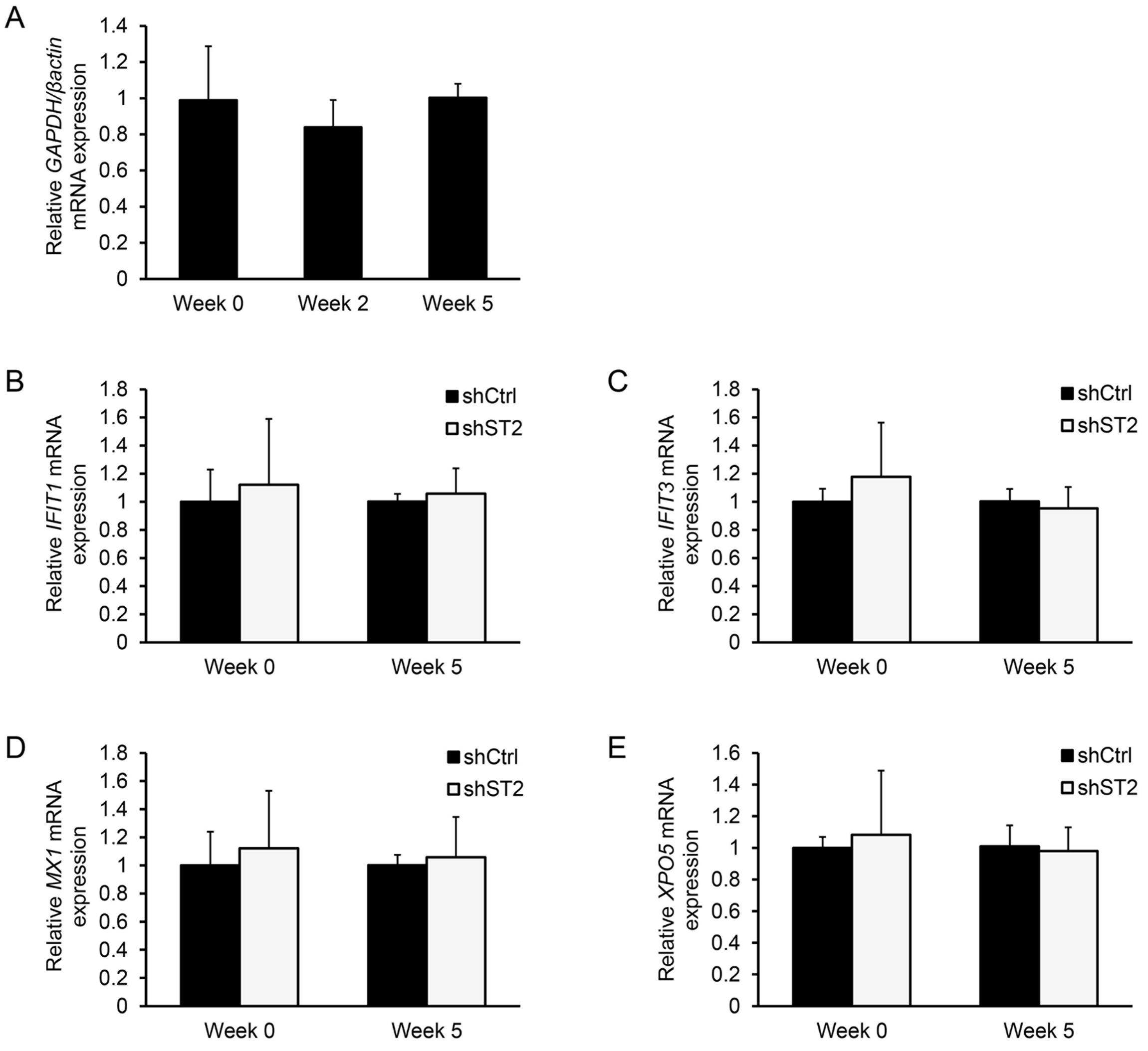

Supplementary Figure S1: Comparison of reference gene expression during ASC adipogenesis and analysis of potential RNAi off- target effects in the stable cell lines. A. ASCs were harvested at the indicated time points of differentiation and subjected to qRT-PCR analysis using primers for the indicated genes. The figure shows the relationship between GAPDH and *β-actin* mRNA expression during the course of this experiment. The results are from four independent experiments, n = 10. **B-E.** ASCs expressing non-silencing shRNA (shCtrl) or shRNA against STAMP2 (shST2) were harvested at the indicated time points of differentiation and subjected to qRT-PCR analysis. The figures show the mRNA expression of *IFIT1* (B), *IFIT2* (C), *MX1* (D), and *XPO5* (E) normalized to the reference gene *GAPDH*. The results are from three independent experiments, n = 8.
